# Changes in disability over time among older working-age adults: Which global and specific limitations are increasing in Germany using the SHARE-data from 2004 to 2015?

**DOI:** 10.1177/20503121231184012

**Published:** 2023-07-08

**Authors:** Johannes Beller, Jelena Epping, Stefanie Sperlich, Juliane Tetzlaff

**Affiliations:** Hannover Medical School, Hannover, Germany

**Keywords:** Disability, functional limitations, morbidity, trends, work

## Abstract

**Objectives:**

Previous studies have observed increasing limitations among the middle-aged, including those aged 40–64, raising the question how healthy work participation has changed. Helping answer this question, we ask: How have general and specific limitations changed in working and non-working adults in Germany?

**Methods:**

We used population-based data of older working-age adults, aged 50–64 years old, from Germany provided by the Survey of Health Aging and Retirement (SHARE) study from 2004 to 2014 (*N* = 3522). Multiple logistic regression analyses were used to study changes in limitations over time.

**Results:**

We found that employment rates generally increased over time, whereas limitation rates mostly increased among participants aged 50–54 and mostly decreased among participants aged 60–64 in both the working and non-working population. Regarding type of disability, increases were more pronounced with movement-related and general activity-related limitations.

**Conclusion:**

Therefore, if the comparatively younger more-limited cohorts age and replace the older less-limited cohorts, a larger part of the working and non-working life might be expected to be spent with limitations in the future, and it seems questionable whether further substantial increases in healthy work participation can be achieved. Further prevention efforts and assistance should be directed at current middle-aged cohorts to improve and maintain their health, including adapting current working conditions to a work force with more limitations.

## Introduction

Disability results from a complex relationship between personal and contextual factors and describes difficulties an individual might have in executing everyday activities. This could include limitations in performing single or multiple activities of daily living (ADL) and instrumental activities of daily living (IADL), which refer to difficulties in self-care like dressing, walking, or shopping. Additionally, disabilities among the middle-aged like those aged 40–64 are often related to difficulties in performing work, such as absenteeism, and early retirement.^1–3^ In large longitudinal studies, disabilities strongly predict increased psychological distress, disease incidence, institutionalization, and mortality.^4–7^ These individual costs of disability are also reflected on a broader economic level: losses in productivity, inability to work, and increased health care expenditures are all associated with disability.^1,2,8,9^ For example, in the United States, national disability-associated health care expenditures were estimated to be about $868 billion in 2015, which represents more than a third of total national health care spending.^10^ Due to possible increases in life expectancy, and shifting demographic composition of the population, this cost is expected to increase even further.^11^

### Disability trends in older working age

Many studies have already analyzed changes over time in limitations in older age groups, aged 65+ years old.^12–16^ However, only few studies have analyzed changes over time in middle-aged adults, which are typically defined as being 40–64 years old, despite the fact that many young and middle-aged adults are also affected by activity limitations.^17–19^ In contrast to many studies showing decreasing limitation trends in older adults, studies in middle-aged adults have often found evidence for increasing limitations across time. In the United States, for example, Zajacova and Montez^20^ found that the prevalence of functional limitations has increased over time. Additionally, they found that this trend might partly result from countervailing forces: The increasing prevalence of psychological distress and income difficulties, as well as the rising incidence of obesity and alcohol use in the United States might have contributed to an increase in midlife disabilities. On the other hand, factors such as the increasing educational attainment and increasing healthy behavioral patterns might have suppressed an even more pronounced increase of these limitations. In Europe, Beller and Epping^17^ focused on generational differences in disability. Using a large sample comprising 15 countries, the authors found that disability seemed to be increasing mostly on the birth cohort level: Younger cohorts born after about 1960 experienced increasing levels of limitations, with especially strong increases over time in limitations in Germany. Several possible factors have been discussed that might explain these cohort differences, but more research is needed to identify the specific causes and mechanisms behind this phenomenon.^17^ To our knowledge, so far studies in middle-aged adults have only used general disability indicators such as the Global Activity Limitation Indicator (GALI) or global ADL and IADL indicators.^21^ However previous studies in older adults have found that disabilities over time vary differentially, with movement-related disabilities, such as walking, standing, stair climbing, sitting, stooping, reaching, grasping, or carrying, seem to increase stronger over time than, for example, self-care or cognitive limitations.^22^ Studies are lacking to examine which specific limitations are increasing over time in middle-aged adults.

Additionally, many studies have explored differences in the prevalence of limitations and trends in limitations between socioeconomic groups.^23^ Similar to other indicators of health, limitation prevalences are found to be higher among the less-educated and the less-affluent.^24–27^ Over time, most studies in Europe pointed to persisting socioeconomic differences in limitations over time.^28,29^ No studies on socioeconomic differences in limitations have been conducted regarding working status, that is, whether working and non-working adults show similar trends in limitations over time. However, previous studies on inequalities between working and non-working adults using self-rated health generally found that inequalities between working and non-working adults have increased over time,^30,31^ suggesting that non-working adults may face increasing barriers to gain and maintain employment. The negative health trends in middle-aged adults together with the growing health-related disparities between working and non-working adults raises the question to what degree the older working-age population (those aged 50–64 years old) can remain economically active over time, especially if negative health trends continue.

### Aims of the current study

In industrialized countries, such as Germany, major changes like the increasing life expectancy, reforms of the welfare system, and the declining health of the middle-aged population have occurred over the past couple of decades possibly affecting the health of the working and non-working populations.^32^ For instance, in Germany, the unemployment benefit has been reduced for long-term unemployment in the last decades, and the number of people who were economically active has increased over time. This development seems to be mainly driven by increasing work force participation of older working-age adults and those with health ailments.^33–35^ Concerns have emerged about how the health of the working and non-working populations can be improved and whether the pension system can remain stable.^36,37^ However, research on time trends in health of working and non-working adults is scarce.

Thus, clearly, more evidence regarding changes in disability over time is needed. The current study contributes to close this gap in the literature. It goes beyond most previous studies by differentiating changes over time regarding specific limitations and by investigating how these changes differ according to employment status and age group. By differentiating between specific types of limitations, more insights into the underlying causes and consequences of disability trends might be gained. For example, some limitations, such as movement-related limitations, may be more strongly associated with certain health problems, such as obesity or a sedentary lifestyle, than others. Moreover, some limitations, such as problems using the toilet, may have more severe impacts on the quality of life and work participation than others. Therefore, knowledge about changes over time regarding specific limitations can help to identify mechanisms driving trends and to design effective targeted interventions to prevent disability and help participants cope with limitations. A population-based sample (*N* = 3522) of the Survey of Health Aging and Retirement in Europe (SHARE) for Germany is used comparing the two time periods 2004 and 2015. Concretely, our main research questions are:

- How have limitations changed in working and non-working adults over time in Germany?
- How long can individuals be expected to work with and without limitations?
- Do these changes in limitations differ by type of limitation and sociodemographic group?

## Methods

### Sample

Data were drawn from the German public release of the SHARE that aims to provide comprehensive data on aging in Europe.^38–40^ Population-based samples of the non-institutionalized population aged 50 and older are provided, collected via multi-stage sampling. Regarding inclusion/exclusion of participants, we used all available data of participations aged 50–64 from the 2004 wave, the first wave of the SHARE study, and all available data of participants aged 50–64 from the 2015 wave, which is the most recent regular wave of the study. In total, *N* = 3543 participants were available from the original SHARE samples in the specific age range which participated either in the 2004 or 2015 wave. We omitted all participants with missing values regarding the key variables (age, gender, limitations, working status) from the sample (*n* = 21; 1% of the original sample). The resulting sample was used as the final sample with a sample size of *N* = 3522 resulted. As a common rule of thumb, one should have at least 10 cases of the least frequent outcome per explanatory variable. In our cases, assuming an average prevalence of 10% for limitations and a maximum of four predictor variables (age, gender, working status, time period) at least 400 cases should be included. Thus, our sample should provide sufficient power for the analyses. Ethics board approval was not required, because we only conducted analyses of completely anonymized SHARE-datasets. Written informed consent was obtained from all subjects before the study.

### Measures

To measure limitations, participants were asked whether they had any difficulty (0 = no difficulty; 1 = has difficulty) with performing everyday activities that they expect to last more than 3 months because of physical, mental, emotional, or memory problems. All specific ADLs and IADLs available via SHARE were included, as stated in the Supplemental Appendix.^41^ Furthermore, to measure GALI, participants were asked to what extent they had been limited because of a health problem in activities people usually do at least for the past 6 months.^42,43^ Participants could respond with “Not limited,” “Limited, but not severely,” and “Severely limited.” The latter two answer categories were combined for the analyses, such that 0 corresponds to “No difficulties” and 1 corresponds to “Limited, but not severely limited or severely limited.” Hence, the analyses differentiate between participants who reported no limitation and those who reported either a severe or non-severe limitation. Working status was operationalized as currently working or not currently working (0 = currently not in general employed or self-employed; 1 = currently in general employed or self-employed). Finally, age and gender were also included. Time period was treated as a categorical variable (0 = 2004; 1 = 2015).

### Statistical analysis

First, descriptive statistics of all variables according to age groups (ages 50–54, 55–59, 60–64), working status (working, non-working), and time period (2004, 2015) are reported. Then, to determine changes across time, multiple logistic regression analyses are conducted predicting limitations via time period for the whole sample controlled for age and gender. In this study, we define trends as changes in the odds of having limitations over time, adjusted for potential confounders. We chose multiple logistic regression as our method to determine changes over time because it allows us to control for age and gender as confounders and to test for interaction effects between time period and other variables such as age group and working status. Then, stratified logistic regression analyses were conducted according to age group and working status, predicting limitations by age, gender, and time period within the aforementioned groups. Differences in time period trends between strata were tested for significance by additionally including interaction terms between the time period and the respective variable (age and/or working status) in the logistic regression analyses. Additionally, in accordance with previous studies, trends in life years individuals can expect to be in paid work (working life expectancy (WLE)) and trends in life years individuals can expect to be healthy, which in this case means being free of GALI, ADL, and IADL limitations and in paid work were calculated (healthy working life expectancy (HWLE)) based on the observed age-specific proportions of working/non-working and disabled/non-disabled populations, conditional on surviving using the Sullivan method.^36,44^ WLE thus represents the number of life years individuals can expect to be in paid work from age 50 onwards irrespective of whether they work in good or bad health. HWLE constitutes a subset of WLE and represents the number of life years to be expected to be in paid work and free of GALI, ADL, and IADL limitations. Accordingly, the difference between WLE and HWLE represents the number of years an individual can be expected to be in paid work while reporting a GALI, ADL, or IADL disability (unhealthy working life expectancy (UHWLE)).

## Results

Overall, as seen in Table 1, participants were on average 57.62 years old (SD = 4.10), with 56% being female and 60% being in active work. About 44% of the sample reported to have a GALI limitation, whereas 40% reported having at least one ADL limitation and 10% reported having at least one IADL limitation. Prevalence of limitations varied widely according to the specific limitation from 1% (making calls) to 24% (kneeling). Furthermore, as also seen in Table 1, limitations were generally higher in older age groups (e.g., GALI_Age50-54_ = 37.8%; GALI_Age55-59_ = 44.7%, GALI_Age60-64_ = 49.3%) and, as seen in Table 2, in non-working adults (e.g., GALI_Working_ = 36.8%; GALI_Non-Working_ = 55.7%). Lastly, as seen in Table 3, limitations partly increased over time on a descriptive level. For example, in 2004 38.2% reported having a GALI limitation, whereas in 2015 49.2% reported having a GALI limitation. Comparatively strong increases in limitations were also observed regarding the ADLs “Getting up,” “Stooping/Kneeling,” and “Reaching Above Shoulder” and regarding the IADL “House Work.” More detailed descriptive statistics can be found in the Supplemental Table A1. Intercorrelations between limitations can be found in the Supplemental Figure A1.

**Table 1.** Disabilities and socio-demographics in German middle-aged adults.

Variables	Overall	Stratified by age		
		50–54	55–59	60–64
*N*	3522	1085	1091	1346
GALI	44.3	37.8	44.7	49.3
ADLs ⩾ 1 (%)	40.2	33.6	40.5	45.3
ADLs, count (mean (SD))	0.99 (1.68)	0.82 (1.59)	0.99 (1.69)	1.11 (1.74)
IADLs ⩾ 1 (%)	9.5	7.7	10.2	10.5
IADLs, count (mean (SD))	0.22 (0.97)	0.16 (0.78)	0.22 (0.90)	0.26 (1.15)
Walking 100 m (%)	4.5	3.2	4.7	5.3
Sitting 2 h (%)	10.4	9.6	12.1	9.8
Getting up (%)	15.8	12.6	16.7	17.6
Climbing flights of stairs (%)	12.9	10.1	11.8	16.0
Climbing flight of stairs (%)	3.7	3.1	3.8	4.1
Stooping/kneeling (%)	23.7	18.5	24.0	27.7
Reaching above shoulder (%)	7.3	6.2	7.7	7.8
Pulling large objects (%)	7.8	7.4	7.2	8.5
Lifting heavy weights (%)	10.2	9.0	9.7	11.7
Picking (%)	2.3	2.3	1.6	2.9
Dressing (%)	3.6	2.2	3.8	4.6
Walking across room (%)	0.7	0.4	0.7	0.9
Bathing (%)	2.0	1.1	2.0	2.6
Eating (%)	0.8	0.3	1.0	1.1
Getting in/out of bed (%)	2.1	1.6	2.2	2.4
Using toilet (%)	0.9	0.6	0.7	1.2
Using map (%)	2.6	2.4	2.7	2.6
Preparing meals (%)	1.1	0.7	0.9	1.6
Shopping (%)	1.4	0.8	1.5	1.9
Making calls (%)	0.4	0.5	0.1	0.7
Taking medications (%)	0.5	0.6	0.5	0.6
House work (%)	4.5	3.6	4.9	4.8
Managing money (%)	1.0	1.2	0.5	1.3
Age (mean (SD))	57.62 (4.10)	52.76 (1.24)	56.96 (1.42)	62.07 (1.42)
Gender (women %)	55.6	59.1	53.0	54.9
Working status (working %)	60.0	76.3	70.0	38.8

**Table 2.** Disabilities and socio-demographics in working and non-working German middle-aged adults.

Variables	Stratified by working status	
	Working	Not working
*N*	2114	1408
GALI	36.8	55.7
ADLs ⩾ 1 (%)	30.8	54.4
ADLs, count (mean (SD))	0.61 (1.19)	1.55 (2.10)
IADLs ⩾ 1 (%)	5.3	15.9
IADLs, count (mean (SD))	0.10 (0.64)	0.40 (1.29)
Walking 100 m (%)	1.3	9.2
Sitting 2 h (%)	6.6	16.2
Getting up (%)	11.2	22.7
Climbing flights of stairs (%)	7.2	21.4
Climbing flight of stairs (%)	1.9	6.4
Stooping/kneeling (%)	17.0	33.8
Reaching above shoulder (%)	4.2	11.9
Pulling large objects (%)	4.1	13.3
Lifting heavy weights (%)	6.4	16.1
Picking (%)	1.1	4.0
Dressing (%)	2.1	6.0
Walking across room (%)	0.2	1.4
Bathing (%)	0.5	4.1
Eating (%)	0.3	1.6
Getting in/out of bed (%)	1.1	3.6
Using toilet (%)	0.3	1.7
Using map (%)	1.5	4.2
Preparing meals (%)	0.4	2.3
Shopping (%)	0.5	2.8
Making calls (%)	0.2	0.8
Taking medications (%)	0.2	1.0
House work (%)	1.8	8.5
Managing money (%)	0.4	2.0
Age (mean (SD))	56.46 (3.68)	59.35 (4.10)
Gender (women %)	52.2	60.7
Working status (working %)	100.0	0.0

**Table 3.** Disabilities and socio-demographics in German middle-aged adults in 2004 and 2015.

Variables	Stratified by time period	
	2004	2015
*N*	1553	1969
GALI	38.2	49.2
ADLs ⩾ 1 (%)	41.1	39.6
ADLs, count (mean (SD))	0.92 (1.59)	1.04 (1.76)
IADLs ⩾ 1 (%)	9.2	9.8
IADLs, count (mean (SD))	0.20 (0.92)	0.23 (1.01)
Walking 100 m (%)	4.1	4.7
Sitting 2 h (%)	10.8	10.2
Getting up (%)	13.8	17.4
Climbing flights of stairs (%)	12.6	13.2
Climbing flight of stairs (%)	4.1	3.4
Stooping/kneeling (%)	21.6	25.4
Reaching above shoulder (%)	6.4	8.0
Pulling large objects (%)	9.0	6.8
Lifting heavy weights (%)	8.2	11.8
Picking (%)	1.5	2.9
Dressing (%)	2.7	4.4
Walking across room (%)	0.7	0.7
Bathing (%)	2.0	1.9
Eating (%)	0.9	0.8
Getting in/out of bed (%)	1.8	2.3
Using toilet (%)	0.8	1.0
Using map (%)	3.2	2.1
Preparing meals (%)	1.2	1.1
Shopping (%)	1.2	1.6
Making calls (%)	0.4	0.5
Taking medications (%)	0.5	0.6
House work (%)	3.3	5.3
Managing money (%)	1.2	0.9
Age (mean (SD))	57.17 (4.34)	57.97 (3.88)
Gender (women %)	53.8	57.0
Working status (working %)	51.6	66.7

**Figure 1.**
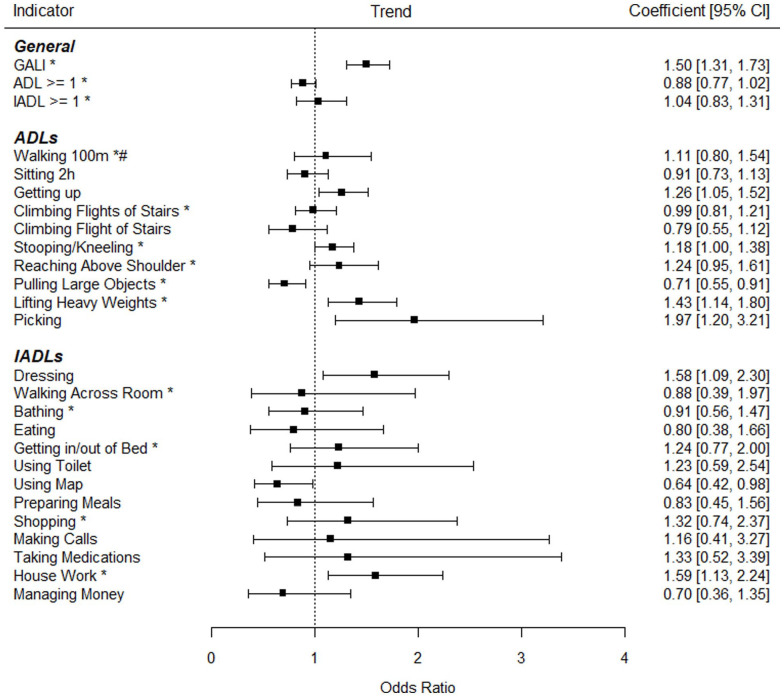
Trend coefficients predicting disabilities in Germany for working and non-working middle-aged adults. *Significant interaction effect of time period with age. ^#^Significant interaction effect of time period with working status. In the interaction analyses, more increasing trends were generally observed in the younger middle-aged adults (e.g., ages 50–54), whereas more decreasing trends were generally observed in the older middle-aged adults (e.g., ages 60–64). Additionally, more increasing trends were observed in the non-working in the case of Walking 100 m. Analyses were adjusted for age and gender.

**Figure 2.**
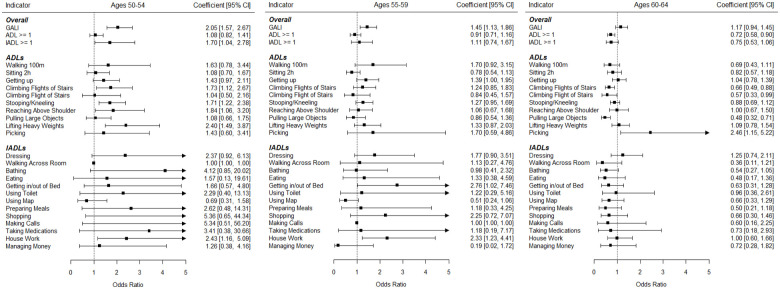
Trend coefficients predicting disabilities in Germany for working and non-working middle-aged adults (left: ages 50–54; middle: ages 55–59; right: ages 60–64). Analyses were adjusted for age and gender.

**Figure 3.**
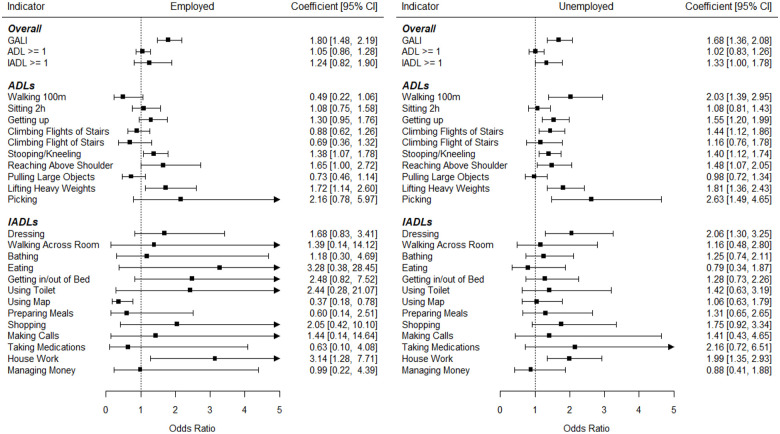
Trend coefficients predicting disabilities in Germany for working (left) and non-working (right) middle-aged adults. Analyses were adjusted for age and gender.

Next, multiple logistic regression analyses were conducted to study trends for limitation indicators adjusted for age and gender. Additionally, interaction effects were examined. As can be seen in Figure 1, limitations significantly increased in the whole sample regarding the following limitations: “GALI” (OR = 1.50), “Getting up” (OR = 1.26), “Stooping/Kneeling” (OR = 1.18), “Lifting Heavy Weights” (OR = 1.43), “Picking” (OR = 1.97), “Dressing” (OR = 1.58), and “House Work” (OR = 1.59). Only the limitations regarding “Pulling Large Objects” (OR = 0.71) and “Using Map” (OR = 0.64) significantly decreased over time. As can also be seen in Figures 2 and 3, several interaction effects according to age but not working status resulted: Increases were stronger in younger adults regarding numerous limitations including the general indicators GALI, ADL ⩾ 1, and IADL ⩾ 1. As seen in Figure 2, increases were especially strong in those aged 50–54 regarding GALI (OR = 2.05), Climbing Several Flights of Stairs (OR = 1.73), Stooping/Kneeling (OR = 1.71), and Lifting Heavy Weights (OR = 2.40). Contrarily, as seen in in the age group 60–64 years old significant decreases in limitations occurred, including ADL ⩾ 1 (OR = 0.72), Climbing Flights of Stairs (OR = 0.66), Climbing Flight of Stairs (OR = 0.57), and Pulling Large Objects (OR = 0.48). Regarding working status, although prevalences were much higher in non-working adults, trends were generally similar to the working participants. Only regarding the “Walking 100 m” limitation, significantly different trends between working status groups were observed, with significantly increasing limitations over time in non-working adults (OR = 2.03) and non-significant decreasing trends in the working sample (OR = 0.49).

**Figure 4.**
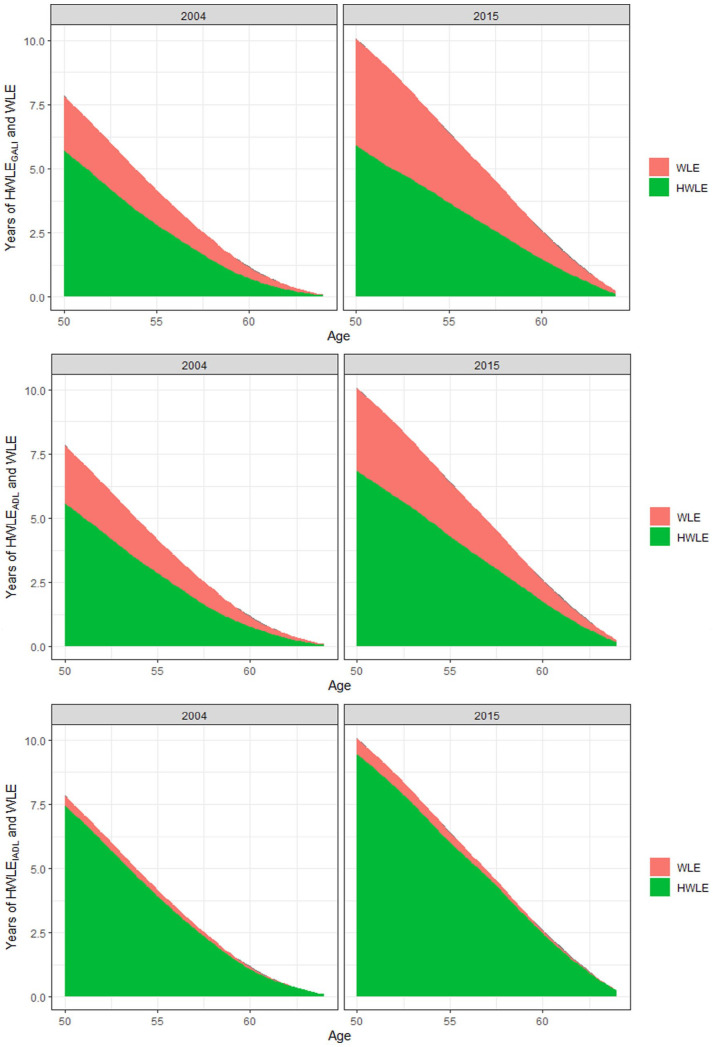
Predicted WLE and HWLE regarding GALI, ADL, and IADL in 2004 and 2015.

**Figure 5.**
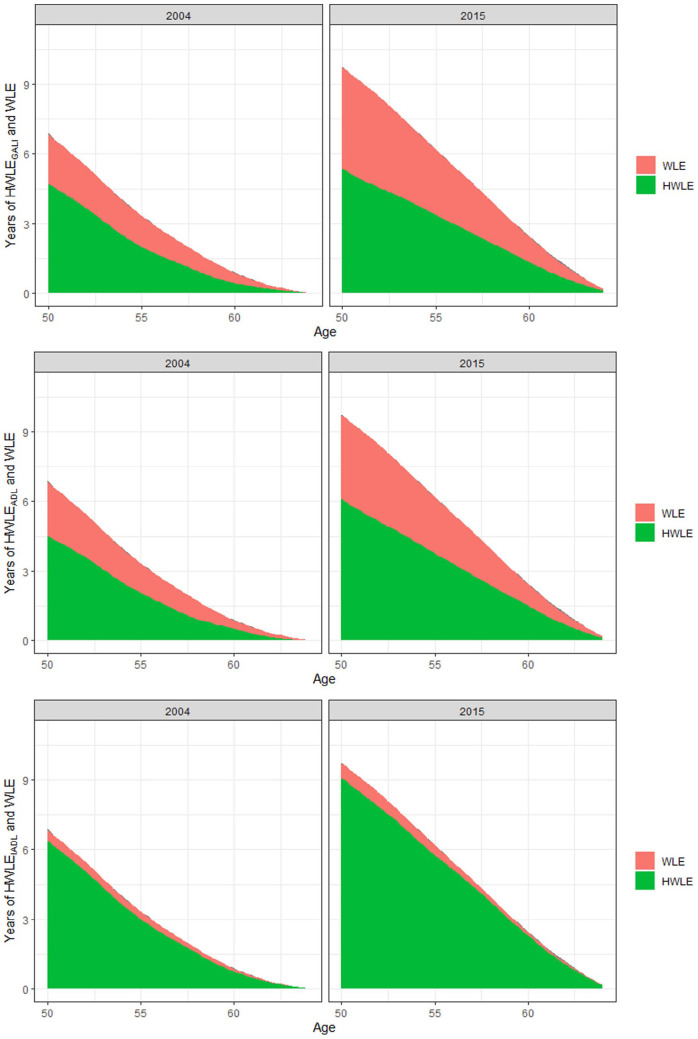
Predicted WLE and HWLE regarding GALI, ADL, and IADL in 2004 and 2015 in women.

**Figure 6.**
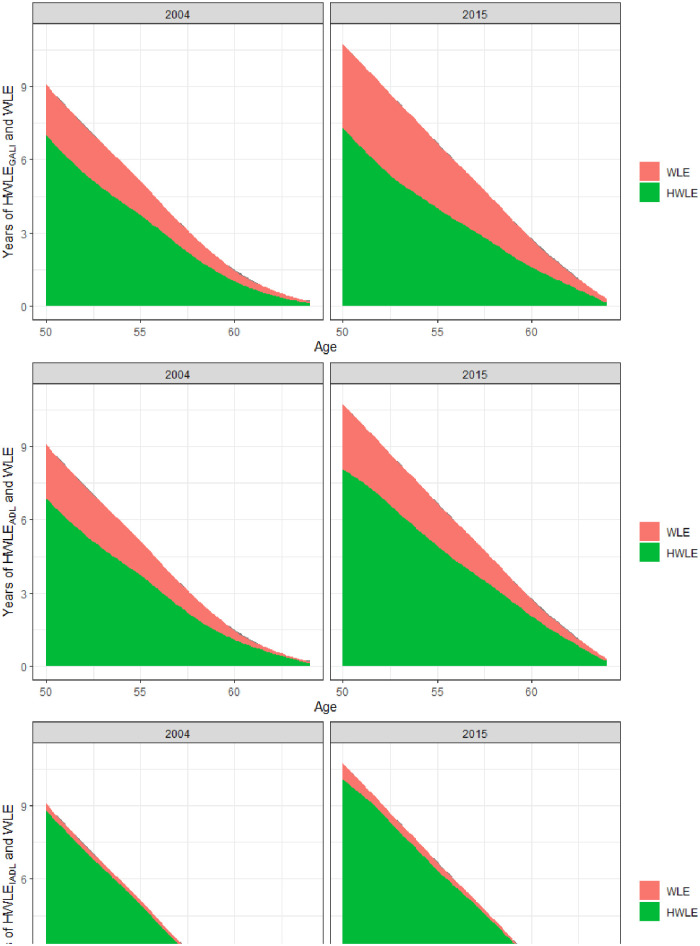
Predicted WLE and HWLE regarding GALI, ADL, and IADL in 2004 and 2015 in men.

Finally, trends in HWLE and WLE are depicted in Figure 4. As can be seen, HWLE generally increased over time, with the strongest increases occurring regarding IADL, followed by ADL and GALI. These increases in HWLE also occurred in each age-group considered, from 50 to 64 years old. At the same time, as also depicted in Figure 4, a substantial increase in WLE occurred over time. Especially in the case of GALI this increase in WLE co-occurred with a substantial increase in UHWLE, especially among the younger age groups considered in the current study aged 50–54 years old. These general trends are replicated across genders as seen in Figures 5 and 6. However, increases in WLE were much more pronounced in women as compared to men. Accordingly, the proportion of time participants can be expected to be working with limitations at age 50 relative to the overall time spent working increased over time from 27% to 41% in the case of GALI but remained stable in the case of ADL (29% versus 32%) and IADL (5% versus 6%).

## Discussion

We examined changes in global and specific disabilities over time among middle-aged working and non-working adults in Germany. We found that both non-working and working adults had high prevalence of limitations, but levels of disabilities were much higher in non-working adults. Over time, more participants were employed and, at the same time, prevalence of many limitations increased. However, trends varied significantly according to age and the limitation considered: Limitations increased most strongly among those aged 50–54 and 55–59 but tended to remain stable or even decreased in those aged 60–64. Additionally, increases were more pronounced for GALI and movement-related limitations. These trends appeared similar in working and non-working adults. In parallel, WLEs increased over time. HWLE also increased for GALI, ADL, and IADL disability across the whole age range considered. However, UHWLE also increased in the case of GALI but remained relatively stable for ADL and IADL. Thus, concretely we found that:

- Limitations changed over time in Germany;
- Changes in limitations depended mainly on the age group considered, with the age groups 50–54 and 55–59 showing mainly increases in limitations over time and age group 60–64 showing decreases over time; additionally, increases in limitations were stronger for movement-related limitations and the GALI;
- WLE increased over time, which results from increases both in HWLE and UHWLE. Thus, more years in the working life from ages 50–64 is expected to be spent in a non-severely (GALI) state, especially regarding movement-related limitations.

These results confirm previous studies. Several studies had suggested that limitations were increasing among the middle-aged population.^17,20^ Supporting these studies, we found some increasing limitations over time. Limitation increases were strongest among those aged 50–54, the youngest age group considered, thus supporting previous research on birth cohort differences in limitations, in which an increasing trend in limitation across generations was found.^17^ However, we also found decreasing trends in limitations among the older age groups considered and generally substantial increases in HWLEs.

Going beyond most previous studies we also differentiated between specific types of limitations, including GALI, ADL, and IADL limitations. To our knowledge only one study had analyzed trends according to different types of limitations and reported that stronger increases were observed regarding movement-related limitations.^22^ These results were also supported in the current study. We found that the strongest significant increases in specific limitations occurred regarding difficulties in lifting heavy weights, doing homework, and reaching above ones shoulder, similar to previous studies where partial decreases in muscular strength were found.^45^ On the contrary, less pronounced changes were generally observed regarding IADL limitations, which are seen as involving a stronger cognitive functioning component.^46^ This is also in accordance with previous studies where a general increase in cognitive functioning was observed over time in older and middle-aged adults.^47–49^ Finally, strong increases in limitations were generally observed regarding the more general GALI item, which refers to “limitations in activities people usually do.” Previous studies have found that in middle-aged adults GALI is strongly related to difficulties with performing work-related activities.^1,2^ At the same time, it has to be noted that significant decreases in limitations were also observed in relatively older adults (ages 60–64) regarding the ADL limitations of climbing stairs and pulling large objects. Thus, over time working-age adults might find it increasingly difficult to cope with work demands, but older adults seem to be less affected by severely limiting disabilities.

Whether trends in limitations differ according to working status had not been systematically studied. In our regression analyses we found similar limitation changes over time for the working and non-working population. This is also in line with previous studies, as most studies in Europe have pointed to mostly persisting differences between groups in limitations over time according to other more frequently studied socioeconomic indicators.^28,29^ However, it must be noted that baseline limitation levels were much higher in the non-working population. Thus, although relative increases were similar, the absolute increases in prevalence were much larger among the non-working population.

One possible explanation for the increase in especially movement-related limitations is the rising prevalence of obesity in Germany, which is a major risk factor for many chronic diseases and especially movement-related limitations.^50^ A possible explanation for the different trends by age group and the similar trends across groups according to working status is that the current study only measures non-severe limitations that might not fully prevent work participation. It is possible that severe limitations show different patterns between age and work groups over time and might affect older age groups more than younger ones. This hypothesis should be tested by further studies.

### Public health implications

These results might be interpreted from both a positive and negative perspective. From a positive viewpoint, the current study has shown that work force participation has increased substantially over time, which is in line with previous survey and statutory health insurance studies.^33,35^ Increased work force participation might not only improve the national economy, and with it, social security systems, but additionally positively affect the well-being of individuals. Indeed, being economically active has been shown to be associated with improved health, especially mental health.^51^ On the contrary, being unemployed has been shown to impair health outcomes.^52–54^ Thus, the trend of being economically active for a longer time period might enable current working-age adults to also be physically and mentally healthy for an extended period of time, thus supporting a potential compression of morbidity.^55^

From a negative viewpoint, ours and several previous studies also observed increasing limitations among middle-aged adults.^17,19,20^ If these increases in limitations prove to be chronic throughout the life course, it is also likely that current middle-aged adults will experience further disabilities and health ailments as they age. Another factor to consider is that the aging of the population likely also affects the demographic composition of the working-age population, which in turn might lead to increased prevalence and severity of limitations in the future on its own. It is thus uncertain whether further increases in work force participation, retirement age, and working lifetime can be achieved, or if alternatively, even decreases in these variables should be expected. Therefore, special prevention efforts and assistance should be directed at current middle-aged cohorts to improve and maintain their health and to support middle-aged individuals with work disability to participate in the workforce. These interventions might include adapting working conditions so that they are more beneficial to a working population with limitations, and to improve health and well-being of the unemployed.^56,57^

Moreover, the increases among movement-related limitations suggest that intervention programs that target these specific limitations could be an effective way to reduce their impact on work ability and quality of life of affected workers.^58^ This could for example include making environmental accommodations like lifts or ramps more widely available or the use of ergonomic work tools. This could also include physical activity interventions that might improve physical functioning.^59,60^ Interventions such as these could also increase the chances of re-employment for those who are out of work due to their limitations.^61^ Thus, by focusing on specific aspects of working-age adults’ functioning, the study contributes to a more nuanced understanding of disability trends and identifies specific areas for practice.

The results should not be taken to suggest that investments should only be made to improve the health of the middle-aged population and not in rehabilitation programs for comparatively older adults. Rather, improving the health of both the current older and middle-aged population via prevention and intervention strategies should be seen as complementary strategies that need to be implemented in face of the challenges of work participation in an aging society. However, our study also highlights the increasing limitations among middle-aged participants, which might necessitate an increased focus on health in the middle-aged cohorts. Nonetheless, more research on the biopsychosocial health, competencies, and needs of the workforce with limitations is needed.

### Limitations

In interpreting the results of the current study, several limitations should be considered. First, the sample did not include institutionalized adults and thus likely underestimates the true level of limitations in the population. Furthermore, we only analyzed self-reported limitations as our dependent variable. Observational methods or physician-based diagnoses might provide outcomes that are less affected by self-report biases. Also, using only two time periods did not allow us to capture possible fluctuations in time trends of limitations. A more detailed analysis with more waves would provide a more comprehensive picture of disability trends among working-age adults. We chose these time points because they were as far apart as possible to capture comparatively longer-term trends in disability. Using more waves would have substantially increased the complexity of the analysis and introduced potential bias given the varying sampling design of the SHARE study. After 2015, the only waves available for Germany at the time of the conceptualization of the study were either related to the COVID-19 pandemic or focused on specific topics that were not suitable for our research question. Since then, several macro-changes might have occurred that have affected the work participation and health of older working-age adults. For example, the COVID-19 pandemic might have negatively affected the health and work participation of those with pre-existing health conditions. Therefore, future research is needed that uses more recent data to replicate and update our findings. Finally, the current study did not analyze the mechanisms behind these trends, which is another potential avenue for future research.

## Conclusion

Notwithstanding these limitations, the current study could show that healthy working life has increased over time. Worryingly, disabilities, especially global and movement-related limitations, have also increased over time in comparatively younger working and non-working adults. This finding questions the possibility of future extensions of healthy working lives, at least with respect to non-severe global activities and physical activity limitations. Further studies should thus replicate and expand upon our results.

## Supplemental Material

sj-docx-1-smo-10.1177_20503121231184012 – Supplemental material for Changes in disability over time among older working-age adults: Which global and specific limitations are increasing in Germany using the SHARE-data from 2004 to 2015? Click here for additional data file. Supplemental material, sj-docx-1-smo-10.1177_20503121231184012 for Changes in disability over time among older working-age adults: Which global and specific limitations are increasing in Germany using the SHARE-data from 2004 to 2015? by Johannes Beller, Jelena Epping, Stefanie Sperlich and Juliane Tetzlaff in SAGE Open Medicine
