# Supplementary material for: Changes in disability over time among older working-age adults: Which global and specific limitations are increasing in Germany using the SHARE-data from 2004 to 2015?
Source: SAGE Open Med. 2023 Jul 8;11:20503121231184012. doi: 10.1177/20503121231184012 (PMC10331346; doi:10.1177/20503121231184012)
Supplement: sj-docx-1-smo-10.1177_20503121231184012 – Supplemental material for Changes in disability over time among older working-age adults: Which global and specific limitations are increasing in Germany using the SHARE-data from 2004 to 2015? [file sj-docx-1-smo-10.1177_20503121231184012.docx]

**APPENDIX**

Included ADLs and IADLs

The following ADLs were included: 1. Walking 100 metres; 2. Sitting for about two hours; 3. Getting up from a chair after sitting for long periods; 4. Climbing several flights of stairs without resting; 5. Climbing one flight of stairs without resting; 6. Stooping, kneeling, or crouching; 7. Reaching or extending your arms above shoulder level; 8. Pulling or pushing large objects like a living room chair; 9. Lifting or carrying weights over 10 pounds/5 kilos, like a heavy bag of groceries; 10. Picking up a small coin from a table.

Additionally, the following IADLs were included: 1. Dressing, including putting on shoes and socks; 2. Walking across a room; 3. Bathing or showering; 4. Eating, such as cutting up your food; 5. Getting in or out of bed; 6. Using the toilet, including getting up or down; 7. Using a map to figure out how to get around in a strange place; 8. Preparing a hot meal; 9. Shopping for groceries; 10. Making telephone calls; 11. Taking medications; 12. Doing work around the house or garden; 13. Managing money, such as paying bills and keeping track of expenses.

Table A1. *Disabilities and Socio-Demographics in German Middle-Aged Adults in 2004 and 2015 According to Working Status and Age-Group*

|  | Working | | | | | | |  |  | Non-Working | |  |  |  |  |
| --- | --- | --- | --- | --- | --- | --- | --- | --- | --- | --- | --- | --- | --- | --- | --- |
|  | Ages 50-54 | | Ages 55-59 | | Ages 60-64 | |  | Ages 50-54 | | Ages 55-59 | | Ages 60-64 | |  |  |
|  | 2004 | 2015 | 2004 | 2015 | 2004 | 2015 | XXX | 2004 | 2015 | 2004 | 2015 | 2004 | 2015 |  |  |
| N | 398 | 430 | 274 | 490 | 129 | 393 |  | 142 | 115 | 171 | 156 | 439 | 385 |  |  |
| GALI | 22.1 | 42.1 | 31.0 | 42.2 | 34.9 | 43.5 |  | 45.1 | 67.0 | 52.0 | 68.6 | 50.6 | 58.4 |  |  |
| ADLs >= 1 (%) | 26.9 | 30.2 | 31.0 | 33.7 | 30.2 | 31.8 |  | 45.1 | 55.7 | 58.5 | 59.0 | 55.4 | 52.7 |  |  |
| ADLs, count (mean (SD)) | 0.42 (0.98) | 0.67 (1.30) | 0.60 (1.26) | 0.71 (1.27) | 0.65 (1.27) | 0.60 (1.08) |  | 1.24 (1.95) | 2.23 (2.60) | 1.42 (1.91) | 2.10 (2.51) | 1.36 (1.82) | 1.52 (2.13) |  |  |
| IADLs >= 1 (%) | 3.0 | 5.6 | 6.2 | 6.9 | 5.4 | 4.6 |  | 14.1 | 24.3 | 15.2 | 21.8 | 13.9 | 14.3 |  |  |
| IADLs, count (mean (SD)) | 0.03 (0.19) | 0.09 (0.53) | 0.09 (0.38) | 0.14 (0.78) | 0.16 (1.17) | 0.10 (0.76) |  | 0.30 (0.94) | 0.70 (1.76) | 0.33 (1.05) | 0.57 (1.45) | 0.35 (1.31) | 0.37 (1.25) |  |  |
| Walking 100m (%) | 1.5 | 0.9 | 2.2 | 1.2 | 2.3 | 0.8 |  | 4.9 | 15.7 | 5.3 | 19.2 | 7.5 | 8.3 |  |  |
| Sitting 2h (%) | 5.3 | 7.4 | 8.0 | 8.8 | 4.7 | 4.1 |  | 18.3 | 21.7 | 22.2 | 18.6 | 12.5 | 14.3 |  |  |
| Getting up (%) | 6.5 | 9.8 | 9.5 | 15.3 | 13.2 | 12.7 |  | 20.4 | 34.8 | 21.1 | 28.8 | 18.2 | 23.4 |  |  |
| Climbing Flights of Stairs (%) | 5.3 | 7.0 | 7.3 | 7.3 | 11.6 | 7.9 |  | 14.1 | 33.9 | 15.2 | 30.1 | 21.4 | 19.7 |  |  |
| Climbing Flight of Stairs (%) | 2.0 | 2.1 | 2.9 | 2.0 | 2.3 | 0.8 |  | 4.9 | 8.7 | 6.4 | 8.3 | 6.2 | 5.7 |  |  |
| Stooping/Kneeling (%) | 10.8 | 18.8 | 15.3 | 20.8 | 17.8 | 17.6 |  | 23.2 | 38.3 | 31.0 | 41.7 | 32.3 | 36.1 |  |  |
| Reaching Above Shoulder (%) | 1.8 | 5.1 | 4.4 | 5.1 | 3.1 | 4.6 |  | 10.6 | 20.0 | 12.3 | 16.7 | 9.1 | 11.2 |  |  |
| Pulling Large Objects (%) | 4.8 | 4.9 | 5.1 | 3.1 | 4.7 | 3.1 |  | 13.4 | 18.3 | 12.3 | 18.6 | 13.9 | 9.4 |  |  |
| Lifting Heavy Weights (%) | 3.8 | 9.8 | 5.1 | 6.5 | 4.7 | 6.6 |  | 9.9 | 23.5 | 13.5 | 23.7 | 12.8 | 17.9 |  |  |
| Picking (%) | 0.8 | 1.6 | 0.4 | 1.2 | 0.8 | 1.5 |  | 4.2 | 7.8 | 2.3 | 3.8 | 1.8 | 6.2 |  |  |
| Dressing (%) | 0.5 | 2.1 | 2.2 | 2.9 | 2.3 | 2.5 |  | 3.5 | 7.0 | 3.5 | 10.3 | 4.6 | 7.5 |  |  |
| Walking Across Room (%) | 0.0 | 0.2 | 0.0 | 0.2 | 0.8 | 0.3 |  | 0.0 | 2.6 | 1.8 | 2.6 | 1.6 | 0.8 |  |  |
| Bathing (%) | 0.0 | 0.7 | 0.4 | 0.6 | 1.6 | 0.5 |  | 1.4 | 6.1 | 4.7 | 6.4 | 4.1 | 3.4 |  |  |
| Eating (%) | 0.0 | 0.2 | 0.0 | 0.8 | 0.8 | 0.3 |  | 0.7 | 0.9 | 2.3 | 1.9 | 1.8 | 1.3 |  |  |
| Getting in/out of Bed (%) | 0.3 | 0.9 | 0.4 | 2.0 | 1.6 | 1.3 |  | 3.5 | 6.1 | 2.3 | 5.8 | 3.4 | 2.6 |  |  |
| Using Toilet (%) | 0.0 | 0.2 | 0.0 | 0.4 | 0.8 | 0.8 |  | 1.4 | 3.5 | 1.8 | 1.9 | 1.4 | 1.6 |  |  |
| Using Map (%) | 1.8 | 0.9 | 2.9 | 1.2 | 2.3 | 1.0 |  | 4.9 | 7.0 | 5.3 | 4.5 | 3.4 | 3.4 |  |  |
| Preparing Meals (%) | 0.3 | 0.2 | 0.7 | 0.4 | 0.8 | 0.3 |  | 0.7 | 4.3 | 1.2 | 2.6 | 2.7 | 2.1 |  |  |
| Shopping (%) | 0.0 | 0.2 | 0.4 | 1.0 | 0.8 | 0.5 |  | 0.7 | 6.1 | 1.8 | 4.5 | 2.7 | 2.6 |  |  |
| Making Calls (%) | 0.0 | 0.2 | 0.0 | 0.2 | 0.8 | 0.3 |  | 0.7 | 2.6 | 0.0 | 0.0 | 0.9 | 0.8 |  |  |
| Taking Medications (%) | 0.0 | 0.2 | 0.4 | 0.2 | 0.8 | 0.3 |  | 0.7 | 3.5 | 0.6 | 1.3 | 0.7 | 0.8 |  |  |
| House Work (%) | 0.5 | 1.9 | 0.7 | 3.5 | 1.6 | 1.8 |  | 7.0 | 16.5 | 6.4 | 15.4 | 5.7 | 7.8 |  |  |
| Managing Money (%) | 0.0 | 0.7 | 0.7 | 0.2 | 0.8 | 0.3 |  | 4.2 | 3.5 | 1.2 | 0.0 | 1.8 | 2.1 |  |  |
| Age (mean (SD)) | 52.33 (1.27) | 53.23 (0.96) | 56.69 (1.41) | 57.08 (1.39) | 61.43 (1.34) | 61.64 (1.31) |  | 52.35 (1.36) | 52.98 (1.18) | 57.06 (1.43) | 56.99 (1.46) | 62.22 (1.43) | 62.54 (1.36) |  |  |
| Gender (Women %) | 53.8 | 59.8 | 43.1 | 51.8 | 37.2 | 54.2 |  | 62.7 | 70.4 | 68.4 | 57.1 | 56.7 | 59.5 |  |  |
| Working Status (Working %) | 100.0 | 100.0 | 100.0 | 100.0 | 100.0 | 100.0 |  | 0.0 | 0.0 | 0.0 | 0.0 | 0.0 | 0.0 |  |  |

Figure A1. *
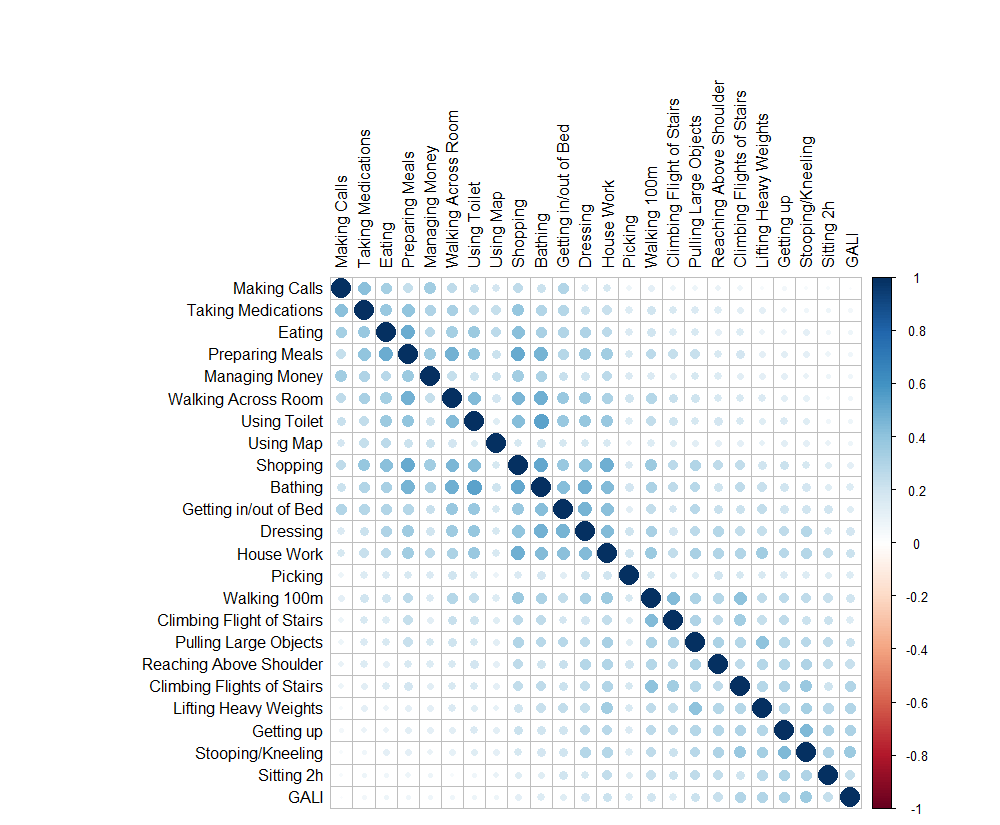
Intercorrelations between limitations in the sample (size of correlations is depicted via size and color of circle).*
